# Validation of kidney transplantation using administrative data

**DOI:** 10.1186/s40697-015-0054-9

**Published:** 2015-05-18

**Authors:** Ngan N Lam, Eric McArthur, S Joseph Kim, Gregory A Knoll

**Affiliations:** Department of Medicine, Division of Nephrology, Western University, London, Ontario Canada; Department of Epidemiology and Biostatistics, Western University, London, Ontario Canada; Institute for Clinical Evaluative Sciences (ICES), Ontario, Canada; Department of Medicine, Division of Nephrology, University of Toronto, Toronto, Ontario Canada; Department of Medicine, Division of Nephrology, Kidney Research Centre, Ottawa, Ontario Canada; Clinical Epidemiology Program, Ottawa Health Research Institute, Ottawa, Ontario Canada; London Kidney Clinical Research Unit, Room ELL-117, Westminster Tower, London Health Sciences Centre, 800 Commissioners Road East, London, Ontario N6A 4G5 Canada

**Keywords:** Administrative data, Canadian Organ Replacement Register (CORR), Kidney transplantation, Validation study

## Abstract

**Background:**

Administrative data are increasingly being used to assess outcomes in kidney transplant recipients.

**Objective:**

To assess the validity of transplant data in healthcare administrative databases compared to the reference standard of information collected directly from transplant centres.

**Design:**

Retrospective cohort study.

**Setting:**

One of three major transplant centres in Ontario (Toronto General Hospital, University Hospital – London, and Ottawa Hospital).

**Patients:**

Recipients who received a kidney-only transplant between 2008 and 2011.

**Measurements:**

For each data source, we identified kidney transplants performed. We calculated the sensitivity and positive predictive value (PPV) of the administrative data for the reference standard data.

**Methods:**

The data collected from transplant centres were compared with data from the Canadian Organ Replacement Register (CORR) database, a hospital procedural code from the Canadian Institute for Health Information Discharge Abstract Database (CIHI-DAD), and provincial physician billing claims from the Ontario Health Insurance Plan (OHIP) database.

**Results:**

During the study period, the three centres reported a total of 1112 kidney transplants performed. The probability of identifying kidney transplant recipients in CORR, CIHI, and OHIP, given they were identified by the transplant centres (sensitivity), was 96%, 98%, and 98% respectively. The probability that the database code correctly identified a transplant recipient (positive predictive value) in CORR, CIHI, and OHIP was 98%, 98%, and 96% respectively.

**Limitations:**

We validated the information from 2008 to 2011 and cannot attest to the reliability of the data beyond the study period. Specifically, we would not regard this as evidence that applies to the earlier years, shortly after the inception of the databases. Secondly, we were unable to distinguish between first and repeat transplantation.

**Conclusions:**

Codes in CORR, CIHI, and OHIP each operate well in the detection of kidney transplant recipients. These data sources can be used to efficiently identify and follow kidney transplant recipients for post-transplant outcomes.

## Abrégé

**Contexte:**

L’utilisation des données administratives tend à se développer dans l’évaluation des résultats chez les greffés du rein.

**Objectifs:**

Évaluer la validité des données sur les greffes contenues dans les bases de données administratives en santé, en les comparant aux informations suivant les normes de référence recueillies directement dans les services de transplantations.

**Type d’étude:**

Étude rétrospective de cohorte.

**Contexte:**

Trois des trois grands services de transplantations de l’Ontario (le Toronto General Hospital, le University Hospital de London et L’Hôpital d’Ottawa).

**Participants:**

Des receveurs qui ont été greffés uniquement du rein entre 2008 et 2011.

**Mesures:**

Nous avons ciblé des greffes qui ont été effectuées pour chacune des sources de données. Nous avons calculé la sensibilité et la valeur prédictive positive (VPP) des données administratives pour les normes de référence en matière d’informations.

**Méthodes:**

Les données recueillies des services de transplantations ont été comparées à celles contenues dans la base de données du Registre canadien des insuffisances et des transplantations d’organes (RCITO), dans un code procédural hospitalier de la Base de données sur les congés des patients de l’Institut canadien d’information sur la santé (BDCP-ICIS), et aux demandes d’indemnisations pour la facturation par les médecins contenues dans la base de données du Régime d’assurance-maladie de l’Ontario (RAMO).

**Résultats:**

Au cours de l’étude, les trois centres ont fait état d’un total de 1112 greffes rénales. Les probabilités d’identifier les receveurs de greffes rénales au RCITO, à l’ICIS et à la RAMQ, considérant qu’ils ont été identifiés par les services de transplantations (sensibilité), étaient respectivement de 96%, de 98%, et de 98%. Les probabilités que le code de la base de données identifie correctement un receveur de greffe (valeur prédictive positive) au RCITO, à l’ICIS et à la RAMQ étaient respectivement de 98%, de 98%, et de 96%.

**Limites de l’étude:**

Nous avons validé l’information de 2008 à 2011, mais ne pouvons attester de la validité des données au-delà de cette période. Plus précisément, l’étude ne pourrait constituer une preuve s’appliquant aux années antérieures, qui suivent de près la mise en place des bases de données. De plus, nous n’avons pas été en mesure de faire une distinction entre les greffes uniques et les greffes répétées.

**Conclusions:**

Les codes du RCITO, de l’ICIS et de la RAMQ sont utiles à la détection des receveurs de greffes. Ces sources de données peuvent être utilisées pour identifier efficacement et retrouver les receveurs de greffes à des fins de suivi de l’issue des greffes.

## What was known before

To identify kidney transplant recipients using administrative databases, information can be obtained from a national transplant registry (Canadian Organ Replacement Register [CORR]), from a hospital procedural code (Canadian Institute for Health Information Discharge Abstract Database [CIHI-DAD]), from a provincial physician billing claim (Ontario Health Insurance Plan [OHIP]), or directly from the transplant centres.

## What this adds

The probability of identifying kidney transplant recipients in CORR, CIHI, and OHIP, given they were identified by the transplant centres (sensitivity), was 96%, 98%, and 98% respectively. The probability that the database code correctly identified a transplant recipient (positive predictive value) in CORR, CIHI, and OHIP was 98%, 98%, and 96% respectively.

## Background

Administrative data is increasingly used in many regions to assess outcomes in kidney transplant recipients (KTR) [1-3]. In Canada, there are multiple databases that can be used to identify KTR. These include local databases maintained by transplant centres, provincial physician billing databases (e.g., Ontario Health Insurance Plan [OHIP]), and national databases (e.g., Canadian Organ Replacement Register [CORR], Canadian Institute for Health Information [CIHI]). Although these datasets are currently being used for research purposes, it remains unknown whether they can reliably identify KTR. We conducted this study to assess the validity of transplant data in healthcare administrative databases compared to the reference standard of information collected directly from transplant centres.

## Methods

Three major transplant centres in Ontario, Canada (London Health Sciences Centre, London; University Health Network, Toronto; and The Ottawa Hospital, Ottawa) provided information on their kidney transplant activity from January 1, 2008 to December 31, 2011. These data were linked to national and provincial healthcare databases at the Institute for Clinical Evaluative Sciences (ICES) using each patient’s encrypted healthcard number. Recipients of simultaneous multi-organ transplants, including kidney-pancreas transplants, were excluded. Information from the transplant centres was considered to be the reference standard. These data were compared to information on KTR in three different healthcare administrative databases. The CORR collects information on all Canadians receiving renal replacement therapy, including dialysis and kidney transplantation. The CIHI Discharge Abstract Database (CIHI-DAD) contains information on diagnostic and procedural information during hospital admissions. The OHIP database contains fee-for-service physician billing claims for both inpatient and outpatient physician services. The codes used to identify KTR from each of the databases are summarized in Table 1.

**Table 1:** **Administrative database codes used to identify kidney transplant recipients**

**Database**	**Kidney transplant code**	**Description**
CORR	*Treatment code:* 171	Acute care hospital, Transplantation, Total care
	*Transplanted organ type code:*	
	10	Kidney/Dialysis
	11	Kidney – Left
	12	Kidney – Right
	18	Kidney – One
	19	Kidney – Two
CIHI	*CCI code:* 1PC85	Transplant, kidney
OHIP	*Fee code:*	
	S435	Kidney transplant
	S434	Kidney re-transplant

*Abbreviations:* CCI, Canadian Classification of Health Interventions; CIHI, Canadian Institute for Health Information; CORR, Canadian Organ Replacement Register; OHIP, Ontario Health Insurance Plan.

For each data source, we compared the number of kidney transplants reported during the study period. We determined the probability of identifying KTR in CORR, CIHI, and OHIP given they were identified by the transplant centres (sensitivity), and the probability the codes in CORR, CIHI, and OHIP correctly identified KTR (positive predictive value [PPV]). For the concordant transplants that were captured by the transplant centres and by the databases, we also assessed the accuracy of the recorded transplant dates. For CIHI, this date was taken as the hospital admission date for the recipient since there is no date specifically associated with hospital intervention codes. The study was approved by the research ethics board at Sunnybrook Health Sciences Centre (Toronto, Ontario, Canada).

## Results

During the study period, the three transplant centres performed 1112 kidney transplants. In comparison, CORR reported 1082 kidney transplants, CIHI reported 1105 kidney transplants, and OHIP reported 1132 kidney transplants (Table 2). All three databases had high sensitivity: CORR 96% (95% confidence interval [CI]: 94% to 97%), CIHI 98% (95% CI: 97% to 99%), and OHIP 98% (95% CI: 97% to 99%). Similarly, all three datasets had high PPV: CORR 98% (95% CI: 98% to 99%), CIHI 98% (95% CI: 97% to 99%), and OHIP 96% (95% CI: 95% to 97%). For the transplants captured by both the transplant centres and the database, the median absolute difference between the recorded transplant dates was 0 days (interquartile range, IQR, 0 to 0) for CORR, 1 day (IQR 0 to 1) for CIHI, and 0 days (IQR 0 to 0) for OHIP.

**Table 2:** **Accuracy of kidney transplant information captured in CORR, CIHI, and OHIP compared to information obtained directly from transplant centres (where the latter served as the reference standard)**

**Database**	**Total number of transplants identified**	**Sensitivity (95% CI)**	**Positive predictive value (95% CI)**
Transplant Centres	1112	-	-
CORR	1082	96% (94% to 97%)	98% (98% to 99%)
CIHI	1105	98% (97% to 99%)	98% (97% to 99%)
OHIP	1132	98% (97% to 99%)	96% (95% to 97%)

*Abbreviations:* CI, confidence interval; CIHI, Canadian Institute for Health Information; CORR, Canadian Organ Replacement Register; OHIP, Ontario Health Insurance Plan.

## Discussion

Provincial and national administrative databases can be used to efficiently follow KTR for post-transplant outcomes provided such databases accurately identify KTR. In this study, three administrative databases, CORR, CIHI, and OHIP, successfully identified most KTR transplanted in the province of Ontario from January 1, 2008 to December 31, 2011.

Moist *et al*. previously performed a validation study of dialysis patients captured in CORR compared to manual chart review and found that demographic information, such as age and sex, had 97% agreement, race had 58% agreement, and primary renal disease had 71% agreement [4]. Co-morbid conditions had sensitivities ranging from 47% (for peripheral vascular disease) to 89% (for hypertension), where the reference standard was patient chart review. The current study extends the validation of the CORR database by assessing the accuracy of the kidney transplant data. To our knowledge, the validation of kidney transplant codes using CIHI or OHIP has not been previously reported. It is reassuring that all three databases had high sensitivity and positive predictive value when compared to the data collected by transplant centres.

There are potential reasons to explain some discrepancies in the information between the transplant centres and the various databases. CORR receives transplantation information from the transplant centres and from provincial organ procurement agencies. There is the possibility of under-reporting by the transplant centres to CORR. Similarly, the hospital-based intervention code from CIHI is abstracted by medical coders who are trained to assign standardized codes on the basis of physician-recorded diagnoses and procedures in a patient’s medical chart [5]. In contrast, the information contained in the OHIP database is from physician billing claims and an over-reporting of cases may have occurred if physicians mistakenly used codes intended for either living donor nephrectomy, kidney auto-transplantation, or the transplantation of other organs.

There are limitations to this study. Although three major transplant centres in Ontario provided center-specific data on their kidney transplant activity, we did not have information from the other three adult transplant centres (Kingston, Hamilton, Toronto – St. Michael’s Hospital). We do not anticipate that the lack of data from these centres would have significantly changed our results. We also validated the information from 2008 to 2011 and, thus, cannot attest to the reliability of the data beyond the study period, particularly in the earlier years at the inception of the databases. For example, CORR began collecting information on organ failure in 1981 and the completeness of the kidney transplant data has likely improved since that time.

## Conclusions

The results of this study suggest that the CORR, CIHI, and OHIP databases have high sensitivity and positive predictive value in identifying KTR compared to information from the transplant centres. These databases can be reliably used to conduct comparative effectiveness and health services research that require the accurate determination of KTR at the population level.

## Consent

The Institute for Clinical Evaluative Sciences (ICES) is a designated prescribed entity under Section 45 of the *Personal Health Information Protection Act* (PHIPA), and as such the need for patient consent is waived.

## Abbreviations

CCI: Canadian Classification of Health Interventions
CI: Confidence interval
CIHI: Canadian Institute for Health Information
CIHI-DAD: Canadian Institute for Health Information – Discharge Abstract Database
CORR: Canadian Organ Replacement Register
ICES: Institute for Clinical Evaluative Sciences
IQR: Interquartile range
KTR: Kidney transplant recipients
OHIP: Ontario Health Insurance Plan
PPV: Positive predictive value
